# Atypical lymphoproliferations associated with germline genetic variants: a report of the 2024 EA4HP/SH lymphoma workshop

**DOI:** 10.1007/s00428-025-04189-0

**Published:** 2025-08-01

**Authors:** Michiel van den Brand, Lívia Rásó-Barnett, Gorana Gasljevic, Olga Balague, Camille Laurent, Maurilio Ponzoni, Ioannis Anagnostopoulos, James R. Cook, Stefan Dirnhofer, Leticia Quintanilla-Martinez, Birgitta Sander, Stefania Pittaluga

**Affiliations:** 1 Pathology-DNA, Location Rijnstate Hospital, Wagnerlaan 55, 6815 AD Arnhem, the Netherlands; 2 Department of Pathology, Radboud University Medical Center, Nijmegen, the Netherlands; 3 The Haematopathology and Oncology Diagnostic Service, Cambridge University Hospitals NHS Foundation Trust, Cambridge, UK; 4 Department of Pathology, Institute of Oncology Ljubljana, Zaloska Cesta 2, 1000 Ljubljana, Slovenia; 5 Faculty of Medicine, University of Maribor, Taborska Cesta 8, Maribor, Slovenia; 6 Department of Pathology, Hospital Clínic, Barcelona, Spain; 7 Department of Pathology, Cancer Institute, Toulouse University Hospital Center, University of Toulouse-Oncopole, Toulouse, France; 8 Vita-Salute San Raffaele University, Hematopathology Diagnostic Area, San Raffaele Scientific Institute, Pathology Unit, Milan, Italy; 9 Institute of Pathology, Julius-Maximilians-University Würzburg, Würzburg, Germany; 10 Department of Pathology and Laboratory Medicine, Cleveland Clinic, Cleveland, OH USA; 11 Institute of Medical Genetics and Pathology, University Hospital Basel, University of Basel, Basel, Switzerland; 12 Institute of Pathology and Neuropathology, Eberhard-Karls-University of Tübingen and Comprehensive Cancer Center, University Hospital Tübingen, Tübingen, Germany; 13 Department of Laboratory Medicine, Division of Pathology, Karolinska Institutet and Karolinska University Hospital, Stockholm, Sweden; 14 Laboratory of Pathology, Center for Cancer Research, Hematopathology Section, National Cancer Institute, NIH, Bethesda, MD USA

**Keywords:** Primary immunodeficiency, Germline mutation, Lymphoma, Pathology

## Abstract

Session 2 of the 2024 European Association for Haematopathology/Society for Hematopathology lymphoma workshop was dedicated to atypical lymphoproliferations in association with germline genetic variants. The first group of cases were lymphoproliferations occurring in the context of primary immunodeficiencies (PID), a heterogeneous group of diseases with increasing incidence and number of different diseases due to better recognition. The workshop contained a spectrum of different PIDs and associated lymphoproliferations with autoimmune lymphoproliferative syndrome, activated phosphoinositide 3-kinase delta syndrome, ataxia-telangiectasia and common variable immune deficiency being the most common. Both children and adults were affected, and the diagnosis of an underlying PID often required a high index of suspicion and correlation with clinical presentation and immunological/ infectious workup. Recognition of a PID allows specific treatment and can influence the interpretation of lymphoproliferations occurring in this context. The spectrum of lymphoproliferations ranged from reactive to overt lymphoma, both EBV-positive and -negative. In a subset of cases, it was very difficult or impossible to establish the boundary between reactive and neoplastic in the context of a PID. The second group represented a heterogeneous group of lymphoproliferations in the context of mutations in germline haematopoietic malignancy risk genes, without associated immunodeficiency. It was often difficult to determine if the genetic defect and the lymphoproliferation were causally related or coincidental, especially if the patient was also treated for non-lymphoid conditions. This is a rapidly evolving field in which future studies are expected to shed more light on the relationship between germline mutations and lymphoid malignancy.

**Supplementary Information:**

The online version contains supplementary material available at 10.1007/s00428-025-04189-0.

## Introduction

Primary immunodeficiencies (PIDs) encompass many diseases, ranging from syndromes lethal in infancy to more subtle abnormalities of the immune system causing symptoms severe enough for medical attention later in life. With the most current classification of the World Health Organization (WHO) and the Primary Immunodeficiencies Committee of the International Union of Immunological Societies (IUIS) recognising 485 distinct primary immunodeficiencies (PIDs) with 375 underlying monogenic defects, familiarity with the full range of PIDs would be challenging in everyday medical practise [1]. Indeed, depending on the affected molecular pathway(s) and cellular component(s) of the immune system, clinical presentation can be highly variable [2]. Clues for an underlying PID are primarily based on clinical and laboratory findings and include recurrent infections, autoimmunity, cytopaenias, a positive family history, allergies and lymphoproliferations [3]. Early recognition of a PID is important as it allows a more appropriate treatment approach, prevents secondary organ damage and is associated with better survival [4].

Considering the prevalence of PIDs of four per 100,000 people in Europe compared to seven per 100,000 of non-Hodgkin lymphomas [5], it is common for haematopathologists to encounter them knowingly or unknowingly. Certain PIDs do result in lesions amenable to biopsy more often than others, particularly those with lymphadenopathy or altered bone marrow function, such as antibody deficiencies and diseases of immune dysregulation. However, as the histomorphological range of patterns is not unique, correlation with clinical, laboratory findings and imaging remains essential to arrive at the correct diagnosis. Furthermore, in the era of increased panel-based sequencing in routine diagnostics, familiarity with the significance of certain somatic variants is also of importance as to correctly recognise this group of disorders, “phenocopies” of PID.

Beyond just achieving the correct histological diagnostic category, establishing the presence of underlying immunodeficiency has practical therapeutic implications as well. Once the immediate treatment needs, e.g. reducing B-cell bulk or treating infections, are met, the diagnosis will inform subsequent treatment strategies as well, avoiding over-treatment as lymphoma, getting the patient to bone marrow transplant before secondary organ damage or balancing treating a possible autoimmune component, which may result in increased infection risk. Genetic counselling and screening of family members are also crucial, both in pre-transplant assessment of the patient as well as in the care and follow up of affected relatives.

Session 2 of the lymphoma workshop of the XXII joint meeting of the European Association for Haematopathology and the Society for Hematopathology in Dubrovnik, Croatia, was devoted to “atypical lymphoproliferations associated with germline genetic variants”. The aim of the panel was to clarify the underlying genetic alteration and type of immunodeficiency and assess best classification of the submitted lesion along the spectrum of lymphoproliferative disorders. The 48 cases received represented a range of PIDs as well as patients with germline mutations in genes possibly associated with an increased risk in haematopoietic malignancies without an associated immune deficiency (Table 1).

**Table 1:** Overview of cases submitted to the workshop

Group	Syndrome, affected gene	Number of patients
Syndrome with autoimmunity	ALPS, *FAS*	5
Immune dysregulation	CVID, underlying mutation unknown	3
	CVID, *TNFRSF13*	1
	APDS1, *PIK3CD*	3
	APDS2, *PIK3R1*	3
	*CTLA4*	1
EBV susceptibility	XLP, *SH2D1A*	3
	XMEN, *MAGT1*	2
	*ITK*	1
	Griscelli syndrome, *RAB27A*	1
	*PRF1*	1
	A20 deficiency, *TNFAIP3*	1
	*TET2*	1
	*RIPK1*	1
DNA repair	*ATM*	5
	*NBS*	1
	*RAG2*	1
CID with syndromic features	Kabuki syndrome, *KMT2D*	1
	Tubular aggregate myopathy, *ORAI/STIM1*	1
Actin-related	Wiskott-Aldrich syndrome, *WAS*	1
	*ARPC1B*	1
	*ACTB*	1
Germline haematopoietic malignancy risk genes	*BRCA1*	1
	*DDX41*	1
	*GATA2*	1
	*PTEN*	1
	*PTPN13*	1
	*SMARCA4*	1
	*TET2*	1
No germline genetic defect	Not applicable	2

*ALPS* autoimmune lymphoproliferative disease, *APDS* activated PI3K delta syndrome, *CID* combined immunodeficiency, *CVID* common variable immune deficiency, *XLP* X-linked lymphoproliferative disease, *XMEN* X-linked MAGT1 deficiency with increased susceptibility to EBV infection and N-linked glycosylation defect

## Lymphoproliferations associated with primary immunodeficiencies (PID)

Lymphoproliferations in the context of PID can form an integral part of the clinical presentation of specific genetic defects, for example, those associated with impaired apoptosis. Alternatively, lymphoproliferations can develop due to a decreased immune surveillance, either with or without the implication of viruses with oncogenic potential. Also, extensive inflammation due to an immune deficiency can induce a tolerogenic microenvironment with simultaneous presence of growth-stimulating cytokines and mutagenic reactive oxygen and nitrogen species.

These lymphoproliferations in the context of a PID span the spectrum of lymphoproliferations occurring in the setting of secondary immunodeficiency, ranging from hyperplasia to monomorphic lesions, with or without EBV positivity [6]. The types of lymphomas (i.e. classic Hodgkin lymphoma and non-Hodgkin lymphoma) are highly variable as is also shown by the large heterogeneity in lymphomas submitted to this workshop session.

### Syndromes with autoimmunity; autoimmune lymphoproliferative syndrome

Five patients were included in the group of syndromes with autoimmunity, all of which fell in the subgroup of autoimmune lymphoproliferative syndrome (ALPS) (Supplementary Table 1). ALPS is a disorder characterised by autoimmunity and lymphoproliferations associated with mutations in genes involved in FAS-mediated apoptosis, with *FAS* itself as the most frequently affected gene. Mutations are most frequently present in the germline, but somatic mutations in *FAS* have been reported in up to 17% of cases [7]. Patients with germline ALPS present at a young age with lymphadenopathy, splenomegaly and autoimmune cytopaenia. The age of five cases submitted ranged from 5 to 44 years, and whilst three of the patients had symptoms already in childhood, two cases were not diagnosed until in their forties when they presented with lymphoproliferations. A definitive diagnosis of ALPS requires (1) the presence of chronic lymphadenopathy and/or splenomegaly which cannot be attributed to infection or malignancy, (2) elevated CD4/CD8 double negative alfa-beta (DN) T cells in the peripheral blood and (3) either the demonstration of a mutation associated with ALPS or the demonstration of defective apoptosis with functional assays [8]. In the absence of a demonstrated mutation or defective apoptosis, a probable diagnosis can be made based on abnormalities in the peripheral blood (elevated sFASL, IL-10, vitamin B12 or IL-18), typical histopathology, autoimmune cytopaenia with elevated IgG and/or the family history.

**Fig. 1:**
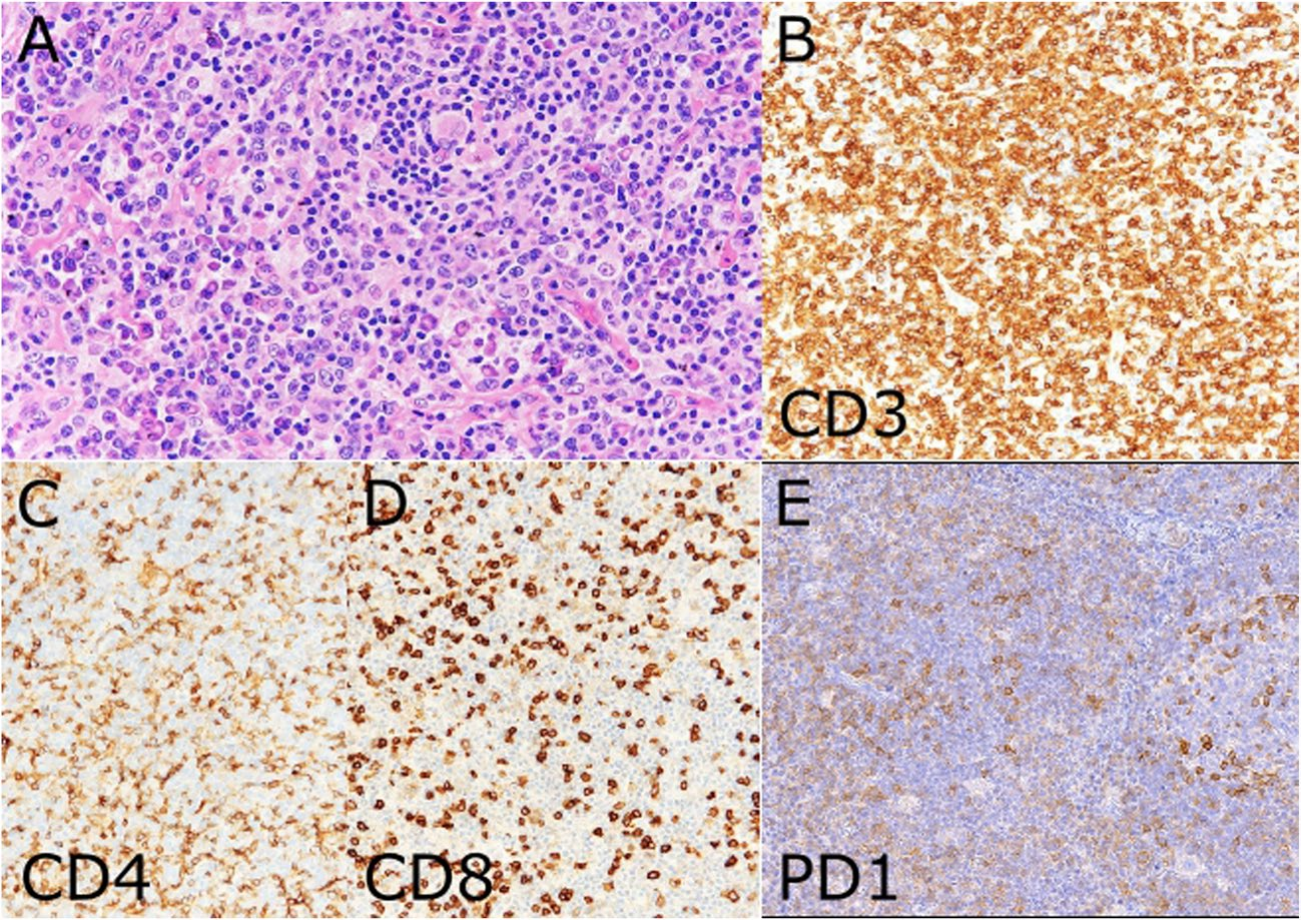
Autoimmune lymphoproliferative syndrome (ALPS). Case LYWS-042 courtesy of C. Chen showed the typical features of ALPS. This cervical lymph node from an 8-year-old boy with extensive lymphadenopathy showed expansion of the paracortex by small lymphocytes, plasma cells and scattered immunoblasts (**A**). Immunohistochemical studies showed a CD4/CD8 double negative T cell population with expression of CD3 (**B**) but not CD4 or CD8 (**C**,** D**). PD1 positive cells can be prominent (**E**) as was illustrated by case LYWS-252 courtesy of F. Climent

A typical presentation of ALPS with the characteristic lymphadenopathy and a demonstrated *FAS* mutation is illustrated by Case LYWS-042 submitted by C. Chen from an 8-year-old boy with extensive lymphadenopathy. The lymphadenopathy in ALPS is characterised by an intact architecture with striking paracortical hyperplasia (Fig. 1) [9]. The paracortex contains variably sized T cells with a high proliferative index with prominent venules and intermixed plasma cells. The B cell compartment can show various changes including florid follicular hyperplasia, progressive transformation of germinal centres and atrophic follicles. Immunohistochemically, the paracortex contains T cells with an increase in DN T cells (Fig. 1). Features of sinus histiocytosis with massive lymphadenopathy (SHML, Rosai-Dorfman disease) can be found in approximately 40% of cases, ranging from isolated S100 + cells with emperipolesis to a confluent histiocytosis [10]. Two cases in the workshop showed features of SHML; LYWS-049 submitted by K. Wilton showed some emperipolesis whilst LYWS-066 submitted by J. Wang showed massive histiocytosis consistent with SHML.

The lymphadenopathy of ALPS with expanded DN T cells can resemble T cell lymphoma, which was underlined by two cases in the workshop. One case was initially considered to be a nodal T-follicular helper cell lymphoma (LYWS-252 presented by F. Climent) in a 40-year-old patient, but when his daughter was diagnosed with ALPS, a genetic investigation was initiated, revealing a *FAS* mutation. The other patient (LYWS-425 submitted by F. Diaz de la Pinta) presented with thrombocytopaenia at the age of 44 and a bone marrow biopsy raised the suspicion T cell large granular leukaemia. However, flow cytometry revealed the presence of DN T cells, and genetic workup showed a *FAS* mutation with a VAF of 53.1%, confirming ALPS. Features that help distinguish between ALPS lymphadenopathy and T cell lymphoma are recognition of DN T cells, preservation of the architecture and the absence of clonality in ALPS.

In addition to the reactive lymphadenopathy, patients with ALPS have an increased risk of lymphoma. These are almost entirely of B cell origin, including classic Hodgkin lymphoma, nodular lymphocyte predominant Hodgkin lymphoma (NLHPL) (nodular lymphocyte predominant B-cell lymphoma, ICC), as well as large B cell lymphoma, comprising T cell and histiocyte-rich large B-cell lymphoma [9, 11–16]. T-cell lymphoma and histiocytic sarcoma have been reported very rarely [9, 17, 18]. The workshop contained one case of NLPHL in the context of ALPS (LYWS-049) with both DN negative T cells, typical for ALPS, as well as CD4/CD8 double positive T cells, associated with NLPHL, detected with flow cytometry.

### Immune dysregulation

This part of the workshop session included a diverse spectrum of diseases with common variable immune deficiency (CVID) and activated PI3K delta syndrome (APDS) being the most common (Supplementary Tables 2–4).

CVID consists of a heterogeneous group of disorders with immunodeficiency and low immunoglobulin levels in which an underlying genetic mutation can only be identified for a subset of patients [19]. Lymphadenopathy is observed in about a quarter of patients [20]. In reactive lymphadenopathy in CVID, the morphology is variable and includes follicular hyperplasia and the presence of granulomas [21–23]. Germinal centres can be irregularly shaped, and progressive transformation of germinal centres is frequently observed. Plasma cells are absent or strongly diminished in number with IgM being the predominant isotype in most cases.

**Fig. 2:**
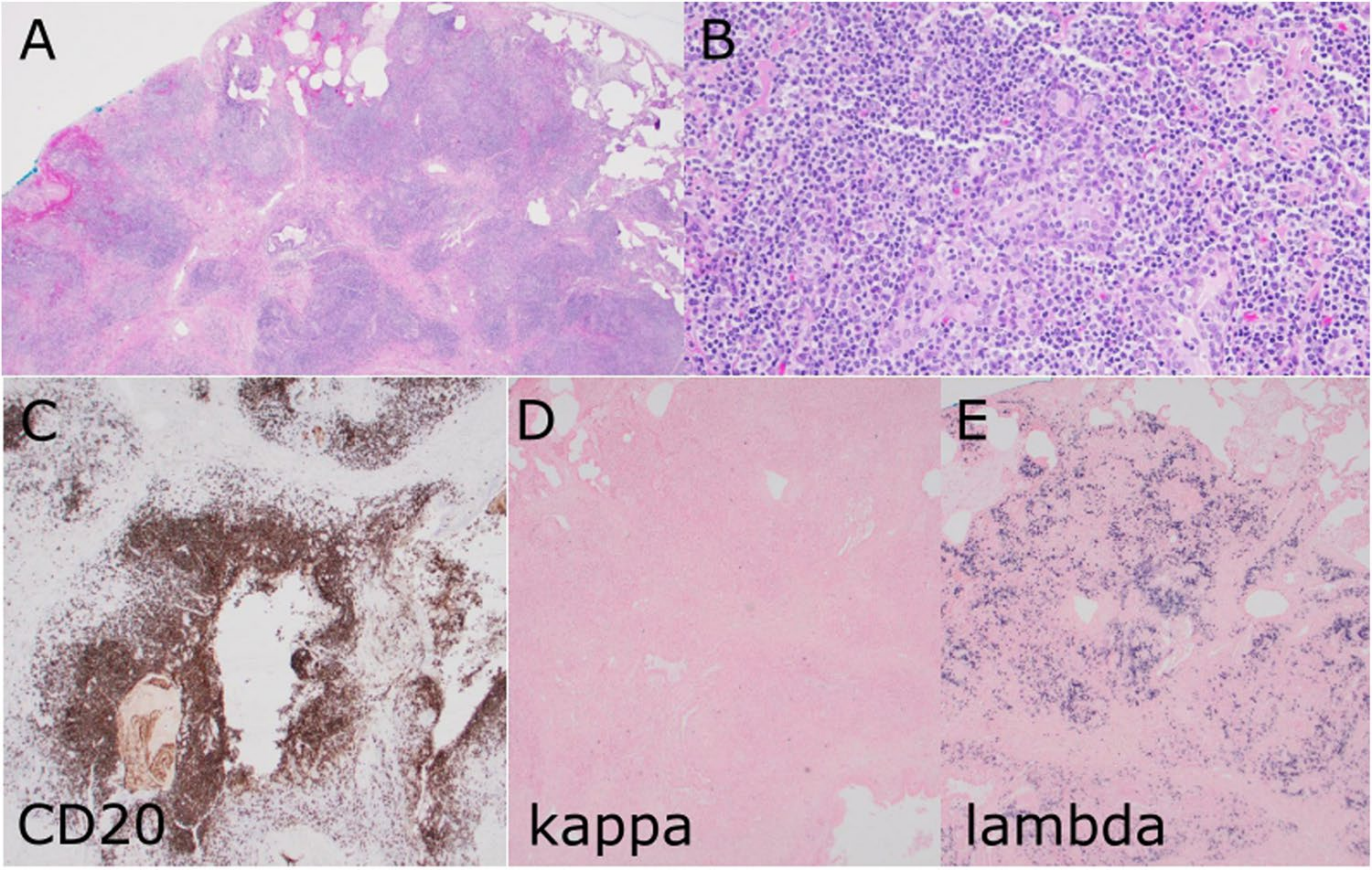
Pulmonary lymphoplasmacytic infiltrate in the context of common variable immune deficiency (CVID). Case LYWS-029 courtesy of H. Tariq represented a wedge resection of the upper lobe of the right lung from a 52-year-old man with a nodular infiltrate of small mature lymphocytes and plasma cells in addition to ill-formed non-necrotising granulomas (**A**,** B**). Immunohistochemistry showed prominent B cells in a nodular distribution (**C**). In situ hybridisation for kappa and lambda light chains (**D** and **E**) showed a clear predominance of lambda light chains. Clonality analysis for IGH showed a clonal result. Although the prominent B cell population with lambda light chain restriction and clonality raised suspicion of an extranodal marginal zone lymphoma, a diagnosis of a reactive lymphoproliferation in the context of CVID was favoured as the architecture was largely retained, and transient clonal populations are known to occur in this context

Patients with CVID have an increased risk of lymphoma which are mostly of B cell origin and which can be EBV positive or negative [24, 25]. Four cases of CVID were included in the workshop of which an underlying defect had only been detected in one patient (LYWS-029 presented by H. Tariq) who carried a germline mutation in *TNFRSF13B* (TACI). These cases illustrated the spectrum from reactive lymphoproliferations to overt lymphoma. Case LYWS-029 showed a lambda-restricted lymphoplasmacytic proliferation in the lung in which B cell clonality was detected, but which was nevertheless considered still consistent with a reactive LPD in the context of CVID (Fig. 2). The distinction between a reactive LPD and marginal zone lymphoma (MZL) in the context of CVID can be very difficult and is to some extent subjective [21, 26]. In this case, the fact that the patient was known to have CVID was a major consideration for making a reactive diagnosis in addition to the underlying architecture, which was largely preserved. Clonal populations in the context of primary immune deficiencies have to be considered carefully as they may be the result of a skewed response to a specific antigen. Case LYWS-162 submitted by S. Naor is from a 10-year-old child with known CVID that first presented with lymphadenopathy with reactive features and monocytoid B cell hyperplasia, but at age 12 developed kappa-restricted nodal MZL with scattered EBV + cells, recurring at the age of 14 with more extensive EBV positivity. This case highlights the progression of the lymphoid proliferation over time in the absence of chemotherapy treatment.

Case LYWS-365 submitted by Y. Liu was a 14-year-old girl who had been diagnosed with EBV-positive classic Hodgkin lymphoma (cHL) 2 years ago and had now developed axillary lymphadenopathy. The biopsy showed a polymorphic LPD, EBV + with follicular and paracortical hyperplasia and aggregates of epithelioid histiocytes. One year later, she developed inguinal lymphadenopathy with similar morphological findings, and a workup for primary immunodeficiency was initiated that resulted in a clinical diagnosis of CVID. Retrospective revision of the original biopsy resulted in reclassification of the original classic Hodgkin lymphoma, as an EBV-positive polymorphic LPD. Case LYWS-329 submitted by L. Frauenfeld was an EBV-positive Burkitt lymphoma (BL) in the setting of CVID. There has been only one other report of BL in the setting of CVID, which was EBV-negative [27].

Activated PI3 kinase delta syndrome (APDS) is an inborn error of immunity caused by mutations in *PIK3CD* (APDS1) or *PIK3R1* (APDS2) which causes overactivation of the PI3K pathway which plays an important role in B and T cell regulation. Patients present with recurrent infections, lymphoproliferations and autoimmunity [28]. The lymphoproliferations occur not only in the lymph nodes and spleen but also in mucosa-associated lymphoid tissues of airways and gastrointestinal tract. Extensive lymphoproliferation in the gastrointestinal tract can lead to intussusception which can be the initial presentation of APDS. In lymph nodes, APDS typically shows a preserved architecture with hyperplastic germinal centres with attenuated to absent mantle zones and aggregates of monocytoid B cells [29]. CD4 + PD1 + T-follicular helper cells are increased both in and outside the follicles. Mucosal lesions endoscopically show nodular hyperplasia. Microscopically, these show features similar to the lymph nodes with proliferation of both B and T cells. APDS patients have an increased risk of lymphoma, comprising cHL Hodgkin lymphoma, diffuse large B cell lymphoma (DLBCL) and MZL [29–31].

**Fig. 3:**
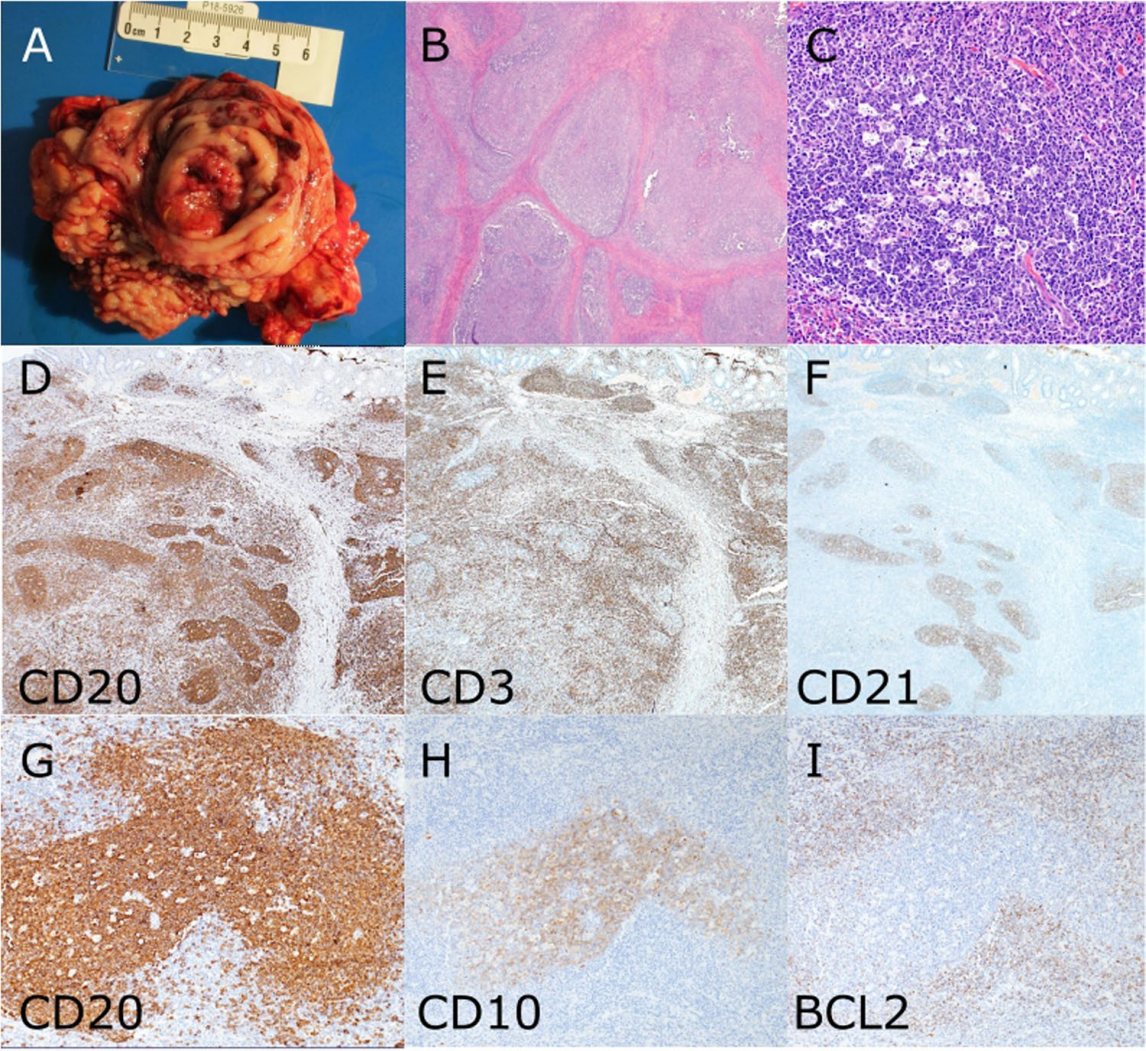
Activated PI3 kinase delta syndrome (APDS). Case LYWS-356 courtesy of E. Mason was a 14-year-old boy with a clinical diagnosis of common variable immune deficiency (CIVD) who presented with a caecal mass which was resected under suspicion of a lymphoma. Macroscopically, a 3.5 cm submucosal mass was found at the appendiceal orifice (**A**). Microscopically, the lesion showed hyperplastic follicles with reactive germinal centres (**B**, **C**). Immunohistochemistry showed a retained architecture with normal distribution of B cells (**D**, CD20), T cells (**E**, CD3) and dendritic meshworks (**F**, CD21). The germinal centres showed a normal immunophenotype with expression of CD20 (**G**) and CD10 (**H**) and negativity for BCL2 (**I**)

The workshop contained three cases each of APDS1 and APDS2. Four patients had a reactive LPD. Prominent proliferation of CD4 + PD1 + T cells was noted in two cases (LYWS-231 submitted by V. Baloda, LYWS-305 submitted by J. Bruneau). Gastrointestinal disease was frequent, ranging from mucosal lymphocytic infiltration detected in biopsies (LYWS-418 submitted by V. Meignin) to presentation with an intestinal mass clinically suspicious for lymphoma (LYWS-231, LYWS-356 presented by E. Mason, Fig. 3). Case LYWS-180 submitted by S. Gibson showed progression from a reactive LPD to a lymphoproliferation considered to be consistent with MZL. In case LYWS-222 submitted by H. Hov, the diagnosis of APDS2 was made at the age of 64 years. This patient presented with a reactive LPD which later progressed to an EBV-negative B cell lymphoma. Case LYWS-214 submitted by M. Stonhill was an 8-year-old girl with lymphoid hyperplasia in the tonsil and cervical lymph nodes in which APDS was in the differential diagnosis, but no mutations were found.

One case of CTLA4 haploinsufficiency was submitted, concerning a 6-year-old boy who presented with bilateral conjunctival masses with prominent B cells with a difficult differential diagnosis between a reactive LPD and MZL (LYWS-438 submitted by J. Enoksson).

### EBV susceptibility

The EBV susceptibility group was heterogeneous and consisted of 11 cases (Supplementary Table 5). Three cases of X-linked lymphoproliferative disease (XLP) were submitted. XLP affects approximately 1–2 per 1 million males and is caused by mutations in *SH2D1A* encoding the SAP protein, which is predominantly expressed in T and NK cells [32]. It is characterised by fulminant EBV infections with haemophagocytic lymphohistiocytosis (HLH), dysgammaglobulinaemia and development of B cell lymphomas, which can be EBV positive or negative [33]. Case LYWS-012 submitted by D. Grier was a 16-year-old boy who presented with fulminant EBV infection in which a rapid diagnosis of XLP could be made by assessment of SAP expression by flow cytometry. In case LYWS-284 submitted by B. Lockhart, a diagnosis of XLP was made incidentally in a 10-year-old boy after genetic screening for mental health problems. Only 1 month after the diagnosis of XLP, the patient developed a high-grade/large B cell lymphoma with 11q aberration. Case LYWS-436 presented by R. Sarro was a 6-year-old boy who developed an EBV-positive DLBCL in which multiple biopsies showed progression of the disease.

There were two cases of X-linked immunodeficiency with magnesium defect, EBV-infection and neoplasia (XMEN), which is caused by mutations in the *MAGT1* gene, resulting in defective glycosylation, thereby causing defective T cell immune responses with increased susceptibility to viral infections including EBV [34, 35]. Case LYWS-119 presented by H. Bharadwaj appeared with multiple viral infections including varicella zoster virus encephalitis and developed an EBV-positive lymphoma consistent with an extranodal MZL. Case LYWS-200 submitted by D. de Jong was a 23-year-old man who was diagnosed with an EBV-negative cHL but who relapsed after 4 years with an EBV positive cHL. Further clinical investigation led to genetic testing which revealed a *MAGT1* mutation.

The remaining cases in the EBV susceptibility group were individual cases with different underlying genetic defects. Two patients presented with HLH with underlying mutations in *RAB27A* (Griscelli syndrome, LYWS-184 submitted by D. Soliman) and *PRF1* (LYWS-288 submitted by M. Donzel). Case LYWS-177 submitted by A. Ku was a 2-year-old girl who developed an EBV-positive cHL in the context of an ITK deficiency. Case LYWS-389 submitted by P. Galera was a 53-year-old man with a germline mutation in *TET2* with mosaicism, who developed a reactive LPD in the lymph node resembling ALPS, followed by a diagnosis of peripheral T cell lymphoma, NOS 1 year later.

For two patients, the relation between the germline mutation and the associated lymphoproliferation was unclear. Case LYWS-299 submitted by A. Cardoni was a 9-year-old girl with a germline mutation in *TNFAIP3* (A20 haploinsufficiency), who developed systemic chronic active EBV disease (CAEBV). The relationship between the A20 haploinsufficiency and CAEBV is unclear, as EBV viraemia has only very rarely been reported in individual cases of A20 haploinsufficiency but not in larger series [36–39]. Case LYWS-293 submitted by L. Bongiovanni was a patient with an indolent EBV-positive T cell proliferation in whom a previously undescribed *RIPK1* mutation was found. Germline mutations in *RIPK1* are associated with cleavage resistant RIP kinase 1 induced autoinflammatory syndrome (CRIA syndrome) [40], but this patient did not show symptoms of CRIA syndrome.

### DNA repair

Four cases of ataxia-telangiectasia (AT) were submitted to the workshop (Supplementary Table 6). AT in its typical form presents in early childhood with cerebellar ataxia, dysarthria, oculocutaneous telangiectasias and immunodeficiency [41]. It is caused by mutations in the *ATM* gene, which encodes a PI3k-related kinase involved in DNA damage repair. Patients with AT have an increased cancer risk, in particular leukaemia and lymphoma before the age of 20 years, but also carcinoma later in life [42]. This was illustrated in the workshop by case LYWS-108 submitted by R. Alamri, which was a girl who developed both a T-lymphoblastic lymphoma/leukaemia and a DLBCL in the first 4 years of life. Two patients developed DLBCL at an age of 6 years (LYWS-285 submitted by B. Lockhart) and 16 years (LYWS-331 presented by I. Montes-Mojarro) in which the latter was preceded by a clonally unrelated lymphoplasmacytic proliferation. Case LYWS-336 submitted by M. Movassaghi was a patient who presented with an epidural mass which was classified as an EBV-negative polymorphic LPD. Case LYWS-037 submitted by M. Brune was 64-year-old man with a lymphoid follicular hyperplasia in the thyroid, who was found to be a carrier of a heterozygous *ATM* mutation.

The two other cases in the group of DNA repair were a patient with Nijmegen breakage syndrome, who developed a T cell lymphoma and T-lymphoblastic leukaemia (LYWS-005 submitted by J. Staniforth) and a patient with atypical lymphoid hyperplasia with a germline mutation in *RAG2* (LYWS-413 submitted by L. Barnea Slonim).

### Combined immunodeficiency with syndromic features

Combined immunodeficiencies with syndromic features are a highly variable group of PIDs. Two cases in this category were submitted to the workshop (Supplementary Table 7). Case LYWS-207 submitted by Y. Hock was a 22-year-old man who was diagnosed in childhood with CHARGE syndrome (coloboma of the eye, heart defects, atresia of choanae, restriction of growth, ear abnormalities), and who now presented with reactive lymphoid hyperplasia. Molecular studies showed a *KMT2D* mutation, indicating that the patient actually had Kabuki syndrome rather than CHARGE syndrome, which is similar clinically, underlining the importance of genetic analysis in making an accurate diagnosis in a patient with PID. Case LYWS-295 submitted by G. Crane was a patient with tubular aggregate myopathy who developed an EBV positive DLBCL of the skin.

### Immunoactinopathies

The actin cytoskeleton is essential for immunologic processes and synapse formation. Accordingly, mutations in different actin-related proteins can cause PID, and these are grouped as immunoactinopathies [43]. Wiskott-Aldrich syndrome (WAS) is the first-described and best known actinopathy, which is caused by mutations in the *WAS* gene, causing thrombocytopaenia, eczema, recurrent infections, autoimmunity and malignancy [43]. Patient with WAS have an incidence of malignancy of approximately 15% before the age of 45, with non-Hodgkin lymphoma being the most common malignancy with the majority being EBV-positive [44, 45]. For the workshop, we received one case of WAS (LYWS-166 submitted by S. Naor, Supplementary Table 8) which was a 1.5-year-old boy with an EBV-negative reactive LPD. Case LYWS-043 submitted by B. Grcar Kuzmanov was a 43-year-old man with an EBV-positive LPD, who was initially considered to have WAS, but genomic sequencing showed a mutation in *ARPC1B*, which encodes a protein involved in actin branching [46]. A third patient (LYWS-178 submitted by A. Ku) suffered from NK-cell deficiency and an EBV-positive LPD due to an underlying mutation in beta actin.

## Germline haematopoietic risk genes

Haematological malignancies can also be caused by germline lesions outside the context of a specific syndrome such as an PID. The availability of next-generation sequencing has shown that germline genetic predisposition to haematological malignancies is more common than previously thought. Similar to PID, the individual germline lesions are rare but together, they are relatively common [47]. Germline predisposition to haematological malignancies is particularly common in myeloid neoplasia in which 5–10% of patients are estimated to have a germline predisposition [48–50]. This has not been so well established for lymphoid malignancies, but germline mutations predisposing to lymphoma have been described in *TP53*, *DICER1*, *KDR*, *KLHDC8B*, *NPAT*, *POT1*, *CHECK2*, *ACAN* and *ETV6* [51]. A difficulty in studying germline predisposition to lymphoma is the fact that co-occurrence of a germline mutation and a diagnosis of lymphoma could be coincidental rather than causal as lymphomas are not uncommon [52].

**Fig. 4:**
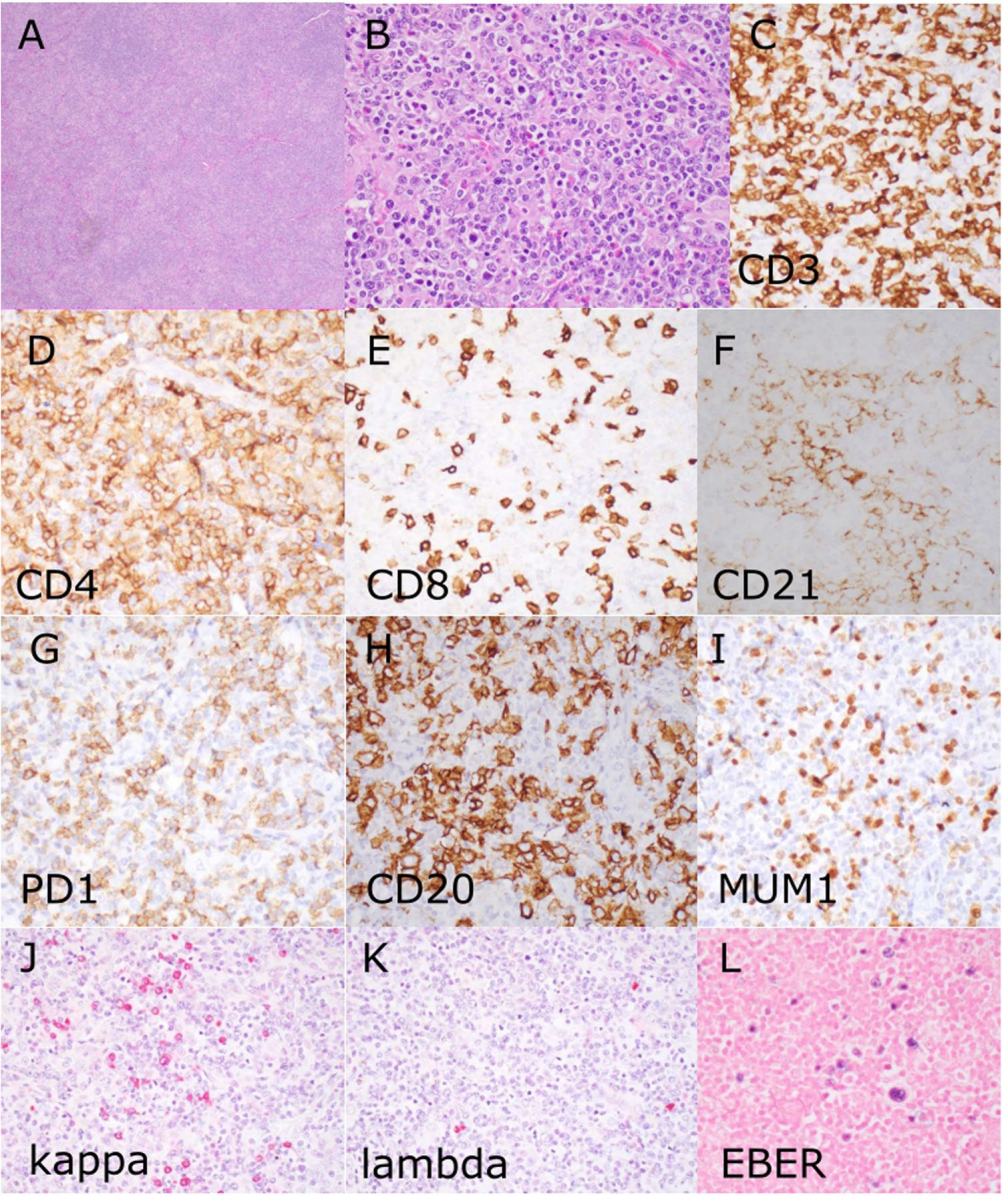
Multiple lymphomas in the context of a germline *TET2* mutation. Case LYWS-189 courtesy of W. Lin was that of a 59-year-old man with a history of nodular lymphocyte-predominant Hodgkin lymphoma/B cell lymphoma who now had lesions in the lung, lymph nodes and spleen. Resection of an axillary lymph node showed loss of normal architecture by a heterogeneous proliferation of lymphocytes with scattered plasmacytoid cells and eosinophils (**A**,** B**). CD3 (**C**) highlighted a prominent T cell component consistent with nodal T-follicular helper cell lymphoma, angioimmunoblastic type, with predominance of CD4 positive T cells (**D** and **E**), expanded dendritic meshworks (**F**, CD21) and expression of T-follicular helper cell markers (**G**, PD1). In addition, a prominent B cell component was present (**I**, CD20) with expression of MUM1 (**J**), monotypic expression of kappa light chains (**K**, **L**) and EBER positivity, consistent with a concurrent EBV-positive B cell lymphoma with plasmacytic differentiation. Next generation sequencing showed a *TET2* mutation with a variant allele frequency (VAF) of 50% which was confirmed to be germline and an additional somatic *TET2* mutation with a VAF of 35%. A similar concurrent T and B cell lymphoma was also found in the lung, but with a different B cell clone which was lambda light chain restricted

Case LYWS-189, presented by W. Lin, was a patient with a germline *TET2* mutation who developed multiple lymphomas (NLPHL, nTFHL, EBV-positive LPD) (Fig. 4, Supplementary Table 9). In line with this case, there have been rare reports in the literature of germline *TET2* mutations causing immunodeficiency and both B and T cell lymphoma [53]. Case LYWS-007 submitted by L. Chen was a patient with a germline mutation in *DDX41* who developed lymphoplasmacytic lymphoma and myelodysplastic syndrome (MDS). *DDX41* germline mutations are associated with myeloid neoplasia, which fits with the fact that the patient had MDS. In addition, the patient had an LPL, which could be a coincidence as larger studies, thus far, do not show a definitively increased lymphoma risk in patients with germline *DDX41* mutations [54].

For the remaining five cases in this group, it was difficult to establish a relationship between the underlying germline mutation and the LPD due to the rarity of the mutation and the possible role of therapy. Case LYWS-280 submitted by F. Ocampo Gonzalez was a 4-year-old girl with a germline *PTPN13* mutation, who developed an EBV-negative DLBCL during treatment with ruxolitinib and dupilumab for a primary atopic disorder. Case LYWS-321 submitted by G. Esteves was a patient with PTEN hamartoma syndrome who developed a T cell LPD, which was more likely caused by prolonged treatment with sirolimus rather than the *PTEN* mutation. Case LYWS-409 submitted by S. Sethi was a patient with a germline *SMARCA4* mutation who was treated with multiple lines of chemotherapy including an EZH2 inhibitor for a small cell carcinoma of the ovary, hypercalcaemic type. She developed a T cell lymphoblastic lymphoma/leukaemia for which it is difficult to establish the contribution of the underlying mutation and the therapy to the development of lymphoma. Case LYWS-450 submitted by B. Shah was a patient with GATA2 deficiency with necrotizing lymphadenitis, which developed whilst under treatment with dasatinib for CML. Another patient (LYWS-075 submitted by N. Zimmermann) with Turner syndrome and a T cell large granular lymphocytic leukaemia had an associated *GATA2* mutation, but this mutation was shown to be somatic rather than in the germline. Case LYWS-130 submitted by A. Chan was a very unusual case of a 49-year-old woman who underwent risk-reducing resection of the ovaries for a germline *BRCA1* mutation. Unexpectedly, a B cell lymphoblastic lymphoma was detected in the ovary but not in the bone marrow.

## Conclusion

This session of the lymphoma workshop highlights the heterogeneity in lymphoproliferations associated with PID and mutations in germline haematopoietic risk genes (Box 1). For the practising pathologist, it is important to be aware that the clinical presentation can be highly variable and that patients can present in adulthood with a less dramatic clinical picture. To increase the chance of identifying these patients, complete information on the clinical presentation, family history, immunological and infectious studies is essential. Although in some specific clinical scenarios, an underlying PID will be included in the differential diagnosis (e.g. recurrent infections, viral encephalitis at young age, autoimmune diseases, persistent reactive lymphoproliferations), in many cases, the presentation is not specific and requires a high index of suspicion (Table 2). Although the morphology of reactive lymph nodes is not per se diagnostic, it can be suggestive of an underlying immune deficiency, and it is important to raise this possibility to prompt genetic testing. Another important issue for pathologists is the presence of clonal rearrangements for IG or TCR. Although detection of a clone can support a diagnosis of lymphoma, it should always be interpreted in the context of morphology, phenotype and clinical presentation. Clonal expansions can be seen when the T cell function fails during which clonal B cell populations may arise. These may change or disappear over time and repeated testing for clonality can be very helpful. With only a single site of disease and a single time point, it can be difficult to determine the importance of a clone, but a cautionary note may help the clinician to guide their approach. The same is true for clonal T cell populations which may arise in response to specific antigens.

**Table:** 

**Box 1**
• There has been a marked increase in the incidence and number of primary immunodeficiencies (PIDs) due to better recognition with next-generation sequencing technologies.
• Identifying PIDs requires a high index of suspicion as the clinical presentation is variable and also includes adults with less pronounced symptoms. Correlation with the clinical context and immunological/infectious workup is essential.
• Early recognition of PIDs is important as this improves prognosis and allows specific treatment.
• Lymphoproliferations in the context of PIDs are heterogeneous and range from reactive to overt lymphoma. EBV can be both positive or negative.
• Some entities can be suspected based on histology, but most do not have specific morphological features.
• In some cases, it is very difficult and, to some extent, subjective to differentiate between a reactive lymphoproliferation or lymphoma in the context of a PID.
• Prominent reactive T cell populations can be present in the context of PIDs and should be differentiated from T cell lymphoma.
• Characterising the range of lymphoid-malignancy-associated germline risk genes is an evolving field. Larger studies are needed to better establish relationships between germline mutations and lymphoid malignancy.

**Table 2:** Clues for the recognition of PID

Clinical presentation	- Recurrent and/or severe infections - Autoimmunity - Allergies - Positive family history - Persistent reactive lymphoproliferations
Morphology	- Range from atrophic germinal centres to florid follicular hyperplasia - Attenuated or absent mantle zones - Emperipolesis
Immunophenotype	- CD4/CD8 double negative T cells - Severely diminished or absent plasma cells - Granulomatous inflammation
Clonality analysis	- Oligoclonal populations - Different clonotypes over time or at different locations

The increasing possibilities for genetic screening are improving the detection of PID and germline mutations associated with haematological malignancies. However, extensive genetic studies are also detecting many more mutations for which it may be difficult to determine whether there is a causal relationship between the mutation and a lymphoproliferation. As genetic screening is becoming more accessible, it is expected that in the coming years, we will obtain a better image of the relationship between underlying germline lesions and associated lymphoproliferations.

## Supplementary Information

Below is the link to the electronic supplementary material.

Supplementary File 1 (DOCX 29.5 KB)

## Data Availability

Not applicable.
