# Supplementary material for: Atypical lymphoproliferations associated with germline genetic variants: a report of the 2024 EA4HP/SH lymphoma workshop
Source: Virchows Arch. 2025 Aug 1;487(2):275–86. doi: 10.1007/s00428-025-04189-0 (PMC12391220; doi:10.1007/s00428-025-04189-0)
Supplement: Supplementary file 1 — (DOCX 29.5 KB) [file 428_2025_4189_MOESM1_ESM.docx]

Supplementary Table 1 - Summary of the clinicopathological features of the 5 cases submitted with a diagnosis of autoimmune lymphoproliferative syndrome (ALPS).

| Case | Age, Sex | Genetic defect | Panel diagnosis | Interesting features | Submitter |
| --- | --- | --- | --- | --- | --- |
| LYWS-42 | 8, M | *FAS* | reactive | Typical ALPS | C. Chen, Rochester, USA |
| LYWS-49 | 43, F | *FAS* | NLPHL (WHO)/ NLPBL (ICC) | CD4-CD8- T-cell of ALPS and CD4+CD8+ T-cells of NLPHL, emperipolesis | K.M. Wilton, Boston, USA |
| LYWS-252* | 40, M | *FAS* | reactive | Initial diagnosis of TFH lymphoma | F. Climent, Barcelona, Spain |
| LYWS-425 | 44, M | *FAS* | reactive | Initial diagnosis of T-LGL | F.J. Diaz de la Pinta, Madrid, Spain |
| LYWS-66 | 5, F | Probable ALPS | Rosai-Dorfman disease | Associated with sinus histiocytosis with massive lymphadenopathy | J. Wang, Philadelphia, USA |

F: female; LYWS: lymphoma workshop; M: male; NLPBL: nodular lymphocyte predominant B-cell lymphoma; NLPHL: nodular lymphocyte predominant Hodgkin lymphoma; TFH: T follicular helper cell; T-LGL: T-large granular lymphocytic leukemia. *Case presented in the workshop.

Supplementary Table 2 - Summary of the clinicopathological features of the 4 cases submitted with a diagnosis of common variable immune deficiency (CVID)

| Case | Age, Sex | Genetic defect | Panel diagnosis | Interesting features | Submitter |
| --- | --- | --- | --- | --- | --- |
| LYWS-162 | 10, F | CVID | 1. Reactive LPD  2. NMZL  3. NMZL, EBV+ | Progression of disease from reactive to lymphoma | S. Naor, Ramat-Gan, Israel |
| LYWS-329 | 49, M | CVID | Burkitt lymphoma,  EBV+ | Burkitt lymphoma rarely reported in CVID | L. Frauenfeld, Tübingen, Germany |
| LYWS-365 | 14, F | CVID | Polymorphic B cell LPD, EBV+ | Earlier diagnosis of classic Hodgkin lymphoma, EBV+ | Y-C. Liu, Memphis, USA |
| LYWS-29* | 52, M | *TNFRSF13B* (TACI) CVID | Reactive LPD | Difficult differential between reactive and lymphoma | H. Tariq, Chicago, USA |

F: female; LPD: lymphoproliferative disorder; LYWS: lymphoma workshop; M: male; NMZL: nodal marginal zone lymphoma. *Case presented in the workshop.

Supplementary Table 3 - Summary of the clinicopathological features of the 6 cases with a diagnosis of activated phosphoinositide 3-kinase delta syndrome (APDS).

| Case | Age, Sex | Genetic defect | Panel diagnosis | Interesting features | Submitter |
| --- | --- | --- | --- | --- | --- |
| LYWS-180 | 27, M | APDS1 | Reactive vs EMZL | Multiple lesions | S. Gibson, Phoenix, USA |
| LYWS-356* | 14, M | APDS1 | Reactive | Caecal mass | E. Mason, Nashville, USA |
| LYWS-418 | 30, M | APDS1 | Reactive | Both lymph node and gastrointestinal lesion | V. Meignin, Paris, France |
| LYWS-222 | 64, M | APDS2 | B-cell lymphoma, NOS | Slow progression of disease, late diagnosis | H. Hov, Trondheim, Norway |
| LYWS-231 | 7, F | APDS2 | Reactive | Multiple lesions, proliferation of PD1+ T-cells | V. Baloda, Pittsburgh, USA |
| LYWS-305 | 3, F | APDS2 | Reactive | Prominent proliferation of PD1+ T-cells | J. Bruneau, Paris, France |

EMZL: extranodal marginal zone lymphoma; F: female; LYWS: lymphoma workshop; M: male; NOS: not otherwise specified. *Case presented in the workshop.

Supplementary Table 4 - Summary of the clinicopathological features of the case submitted with a diagnosis of CTLA4 haploinsufficiency

| Case | Age, Sex | Genetic defect | Panel diagnosis | Interesting features | Submitter |
| --- | --- | --- | --- | --- | --- |
| LYWS-438 | 6, M | *CTLA4* haploinsufficiency | Marginal zone lymphoma vs. reactive lymphoproliferative disease | Bilateral conjunctival lesions | J. Enoksson, Lund, Sweden |

LYWS: lymphoma workshop; M: male.

Supplementary Table 5 - Summary of the clinicopathological features of the 11 cases associated with EBV susceptibility

| Case | Age, Sex | Genetic defect | Panel diagnosis | Interesting features | Submitter |
| --- | --- | --- | --- | --- | --- |
| LYWS-12 | 16, M | *SH2D1A* (XLP) | Fulminant EBV+ LPD | Rapid diagnosis with SAP flow cytometry | D. Grier, Cincinatti, USA |
| LYWS-284 | 10, M | *SH2D1A* (XLP) | HGBCL/LBCL-11q | Mutation found coincidentally | B. Lockhart, Philadelphia, USA |
| LYWS-436* | 6, M | *SH2D1A* (XLP) | DLBCL, EBV+ | Multiple samples 🡪 progression of disease | R. Sarro, Locarno, Switzerland |
| LYWS-119* | 19, M | *MAGT1* (XMEN) | EBV+ B-cell lymphoma with plasmacytic differentiation | Typical presentation with multiple infections | H. Bharadwaj, Boston, USA |
| LYWS-200 | 23, M | *MAGT1* (XMEN) | 1. cHL, EBV- 2. Polymorphic LPD, EBV+ | Diagnosis made after relapse | D. De Jong, Amsterdam, the Netherlands |
| LYWS-177 | 2, F | *ITK* | cHL, EBV+ | Infectious complications of therapy | A. Ku, New York, USA |
| LYWS-184 | 21, M | *RAB27A* (Griscelli syndrome) | HLH | Presentation at older age | D. Soliman, Doha, Qatar |
| LYWS-288 | 17, F | *PRF1* | HLH | Lack of immunohistochemical perforin staining | M. Donzel, Lyon, France |
| LYWS-299 | 9, F | *TNFAIP3* (A20) | CAEBV | Uncertain relationship between *TNFAIP3* mutation and CAEBV | A. Cardoni, Rome, Italy |
| LYWS-389 | 53, M | *TET2* (mosaic) | 1. Reactive, ALPS-like 2. PTCL, NOS | EBV negative | P. Galera, New York, USA |
| LYWS-293 | 32, F | *RIPK1* | Indolent ENKTL | Relevance of *RIPK1* mutation uncertain | L. Bongiovanni, Milan, Italy |

ALPS: autoimmune lymphoproliferative syndrome; CAEBV: systemic chronic active EBV disease; cHL: classical Hodgkin lymphoma; DLBCL: diffuse large B-cell lymphoma; ENKTL: extranodal NK/T-cell lymphoma; F: female; HGBCL: high-grade B-cell lymphoma; HLH: haemophagocytic lymphohistiocytosis; LBCL: large B-cell lymphoma; LPD: lymphoproliferative disorder; LYWS: lymphoma workshop; M: male; PTCL, NOS: peripheral T-cell lymphoma, not otherwise specified; SAP: SLAM-associated protein. *Case presented in the workshop.

Supplementary Table 6 - Summary of the clinicopathological features of the 7 cases associated with defects in DNA repair

| Case | Age, Sex | Genetic defect | Panel diagnosis | Interesting features | Submitter |
| --- | --- | --- | --- | --- | --- |
| LYWS-108 | 1, F | *ATM* | T-LBL, DLBCL | Multiple lymphomas | R. Alamri, Pittsburgh, USA |
| LYWS-331* | 16, M | *ATM* | Lymphoplasmacytic proliferation, EBV- | Patient later developed DLBCL (not clonally related) | I. Montes-Mojarro, Tübingen, Germany |
| LYWS-285 | 6, F | *ATM* | DLBCL, EBV+ | Development of chronic EBV after treatment | B. Lockhart, Philadelphia, USA |
| LYWS-336 | 22, F | *ATM* | Polymorphic LPD, EBV- | Presentation with epidural mass | M. Movassaghi, Los Angeles, USA |
| LYWS-37 | 64, M | *ATM* carrier | Reactive lymphoproliferation | Thyroid follicular hyperplasia | M. Brune, Basel, Switzerland |
| LYWS-5 | 10, M | *NBS* | Near-ETP ALL | Earlier diagnoses of T-cell lymphoma and T-LBL | J. Staniforth, Cambridge, United Kingdom |
| LYWS-413 | 31, F | *RAG2* | Atypical lymphoid hyperplasia | Reactive vs. MZL | L. Barnea Slonim, London, United Kingdom |

DLBCL: diffuse large B-cell lymphoma; F: female; LYWS: lymphoma workshop; LPD: lymphoproliferative disorder; M: male; MZL: marginal zone lymphoma; Near-ETP-ALL: near-early T-cell precursor acute lymphoblastic leukaemia; T-LBL: T-lymphoblastic leukemia/ lymphoma. *Case presented in the workshop.

Supplementary Table 7 - Summary of the clinicopathological features of the 2 cases of combined immune deficiency with syndromic features

| Case | Age, Sex | Genetic defect | Panel diagnosis | Interesting features | Submitter |
| --- | --- | --- | --- | --- | --- |
| LYWS-207 | 22, M | *KMT2D* (Kabuki syndrome) | Reactive LPD | Initial diagnosis of CHARGE syndrome | Y. Hock, Birmingham, United Kingdom |
| LYWS-295 | 69, M | *ORAI1/ STIM1* (Tubular aggregate myopathy) | DLBCL, EBV+ | 10 years prior also DLBCL, EBV+ | G. Crane, Cleveland, USA |

DLBCL: diffuse large B-cell lymphoma; LPD: lymphoproliferative disorder; LYWS: lymphoma workshop; M: male.

Supplementary Table 8 - Summary of the clinicopathological features of the 3 cases associated with immunoactinopathies

| Case | Age, Sex | Genetic defect | Panel diagnosis | Interesting features | Submitter |
| --- | --- | --- | --- | --- | --- |
| LYWS-166 | 1.5, M | *WAS* | Reactive LPD, EBV- | Differential diagnosis with B-cell lymphoma | S. Naor, Ramat-Gan, Israel |
| LYWS-43 | 27, M | *ARPC1B* | EBV+ LPD | Initially considered to be WAS | B. Grcar Kuzmanov, Ljubljana, Slovenia |
| LYWS-178 | 30, M | *ACTB* (NKD) | EBV+ LPD, Hodgkin-like | NK-cell deficiency, presentation at older age | A. Ku, New York, USA |

LPD: lymphoproliferative disorder; LYWS: lymphoma workshop; M: male; NKD: NK-cell deficiency; WAS: Wiskott-Aldrich syndrome.

Supplementary Table 9 - Summary of the clinicopathological features of the 7 cases associated with germline haematopoietic malignancy risk genes

| Case | Age, Sex | Genetic defect | Panel diagnosis | Interesting features | Submitter |
| --- | --- | --- | --- | --- | --- |
| LYWS-189* | 59, M | *TET2* | TFH lymphoma; EBV+ B-cell lymphoma | Multiple lymphomas | W. Lin, New York, USA |
| LYWS-7 | 65, M | *DDX41* | LPL | Patient also developed MDS | L. Chen, Auckland, New Zealand |
| LYWS-280 | 4, F | *PTPN13* | DLBCL, EBV- | Uncertain association between *PTPN13* mutation and lymphoma | F. Ocampo-Gonzalez, New York, USA |
| LYWS-321 | 24, M | *PTEN* | T-cell LPD | T-LPD most likely due to sirolimus treatment | G. Esteves, Lisbon, Portugal |
| LYWS-409 | 38, F | *SMARCA4* | T-LBL | Uncertain association between *SMARCA4* mutation and lymphoma | S. Sethi, New York, USA |
| LYWS-450 | 40, M | *GATA2* | Necrotising lymphadenitis | Also CML with dasatinib treatment | B. Shah, Philadelphia, USA |
| LYWS-130 | 49, F | *BRCA1* | B-LBL | Incidental finding, *BCL2* rearrangement | A. Chan, New York, USA |

B-LBL: B-lymphoblastic lymphoma; CML: chronic myeloid leukaemia; DLBCL: diffuse large B-cell lymphoma; F: female; LPL: lymphoplasmacytic lymphoma; LYWS: lymphoma workshop; M: male; MDS: myelodysplastic syndrome; TFH: T follicular helper cell; T-LBL: T-lymphoblastic leukaemia/ lymphoma; T-LPD: T-cell lymphoproliferative disorder. *Case presented in the workshop.

Supplementary Table 10 - Summary of the clinicopathological features of the 2 cases with no underlying germline genetic defect

| Case | Age, Sex | Genetic defect | Panel diagnosis | Interesting features | Submitter |
| --- | --- | --- | --- | --- | --- |
| LYWS-75 | 30, F | *GATA2* (somatic) | T-LGL | Turner syndrome, pancytopenia | N. Zimmermann, Cincinatti, USA |
| LYWS-214 | 8, F | none | Marginal zone hyperplasia | Differential diagnosis of APDS but no mutation | M. Stonhill, Massachusetts, USA |

APDS: activated phosphoinositide 3-kinase delta syndrome; F: female; LYWS: lymphoma workshop; T-LGL: T-large granular lymphocytic leukaemia.
